# Supplementary material for: Integrated Microbiome–Metabolome Analysis and Functional Strain Validation Reveal Key Biochemical Transformations During Pu-erh Tea Pile Fermentation
Source: Microorganisms. 2025 Aug 8;13(8):1857. doi: 10.3390/microorganisms13081857 (PMC12388847; doi:10.3390/microorganisms13081857)
Supplement: Supplementary file 1 [file microorganisms-13-01857-s001.zip › microorganisms-3762391-supplementary.pdf]

Integrated Microbiome–Metabolome Analysis and Functional Strain Validation Reveal Key Biochemical Transformations During Pu-erh Tea Pile Fermentation

This file includes:

Tables S1 to S2

Figs. S1 to S4

Supplementary Tables

Supplementary Table 1: Details of Yunnan Little Brown Pu-erh tea samples.

Time (days)	Sample	Fermentation period
0D	BPT/BF_0D	Pre-fermentation
1D	BPT/BF_1D	
2D	BPT/BF_2D	
3D	BPT/BF_3D	1st fermentation stage
5D	BPT/BF_5D	
6D	BPT/BF_6D	
10D	BPT/BF_10D	
12D	BPT/BF_12D	2nd fermentation stage
14D	BPT/BF_14D	
18D	BPT/BF_18D	
20D	BPT/BF_20D	3rd fermentation stage
22D	BPT/BF_22D	
26D	BPT/BF_26D	4th fermentation stage
28D	BPT/BF_28D	5th fermentation stage
30D	BPT/BF_30D	End-point

Notes: BPT indicates the name of the sample for the testing of prokaryotic microorganisms, and BF indicates the name of the sample for the testing of eukaryotic microorganisms.

Supplementary Table 2: Primers used in this study.

Primer name	Sequence
338F	ACTCCTACGGGAGGCAGCA
806R	GGACTACHVGGGTWTCTAAT
ITS1F	GGA AGTAAA AGTCGTAACAAGG
ITS2R	GCTGCGTTCTTCATCGATGCT

Supplementary Figures

Supplementary Figure 1: The fermentation process of Little Brown Pu-erh tea.

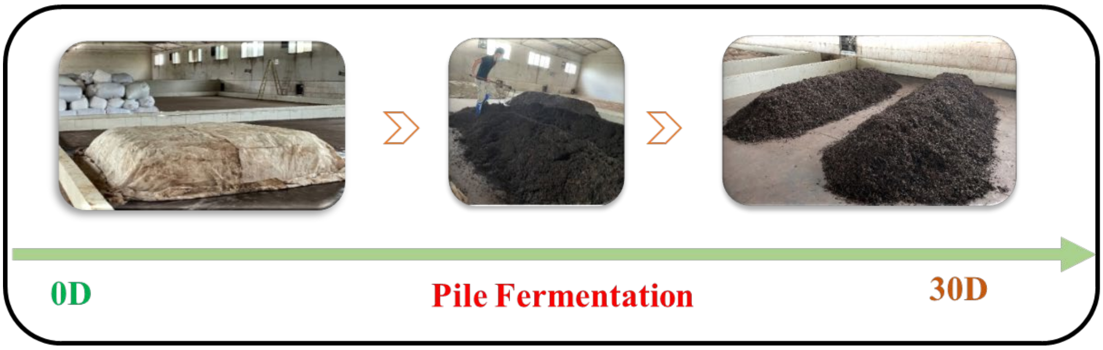

Supplementary Figure 2: A schematic diagram of sample collection (AB=AC=AD=AE=1 m).

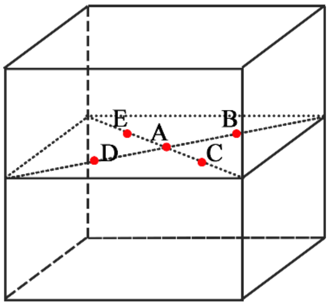

Supplementary Figure 3: Morphological and microscopic features of culturable microbial strains isolated from Pu-erh tea pile fermentation.

Strain	Colony morphology	Gram staining	Strain	Colony morphology	Gram staining
Bacillus haynesii	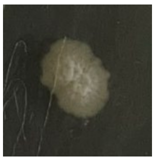	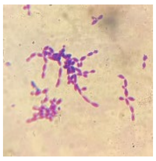	*Staphylococcus lloydii*	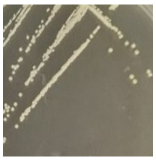	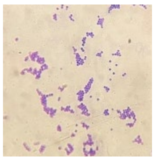
Staphylococcus gallinarum	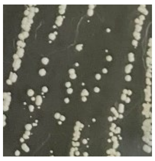	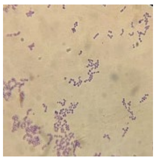	*Pluralibacter gergoviae*	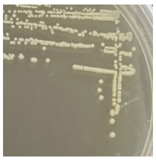	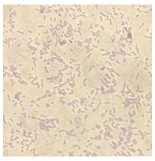
Bacillus licheniformis	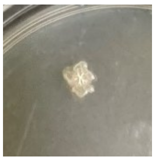	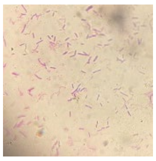	*Klebsiella pneumoniae*	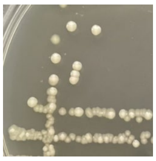	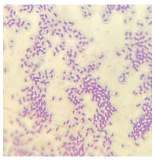
Aeromonas caviae	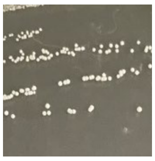	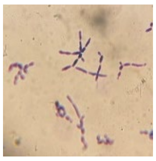	*Priestia filamentosa*	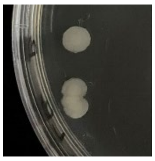	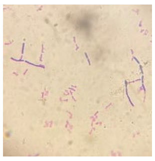
Priestia aryabhattai	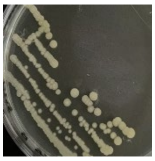	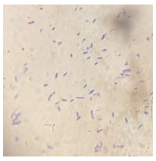	*Enterococcus faecium*	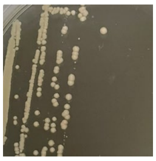	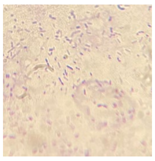
Curtobacterium citreum	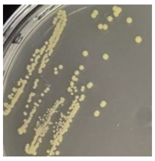	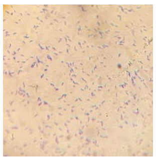	*Bacillus subtilis*	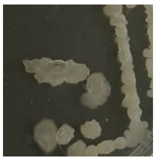	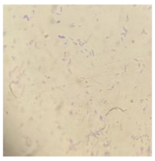
Rothia halotolerans	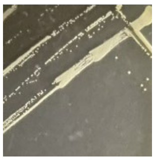	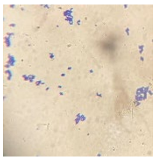	*Pseudomonas guariconensis*	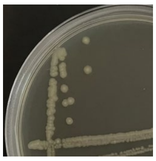	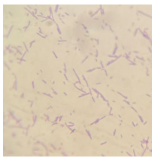
Lysinibacillus macroides	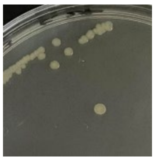	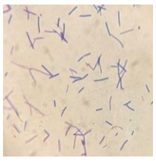	*Mammaliicoccus sciuri*	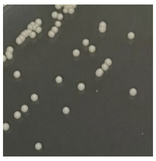	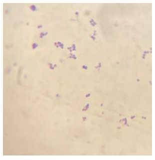
Bacillus amyloliquefaciens	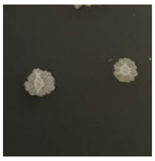	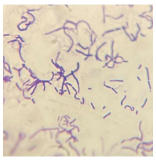			

Supplementary Figure 3 (Continued)

Strain	Top view	Bottom view	Strain	Top view	Bottom view
Rhizomucor pusillus	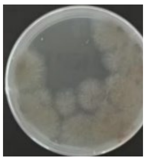	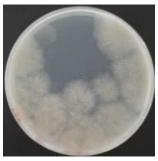	*Aspergillus costaricensis*	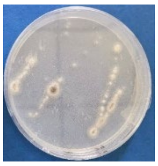	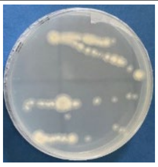
Cyberlindnera rhodanensis	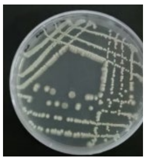	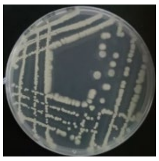	*Hamigera fusca*	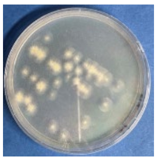	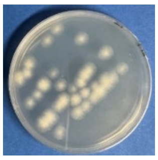
Blastobotrys adeninivorans	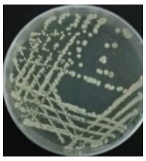	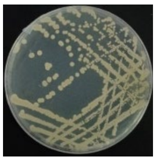	*Hamigera insecticola*	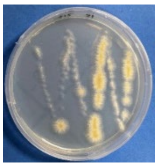	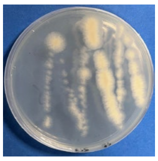
Aspergillus tubingensis	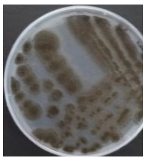	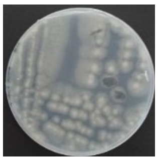	*Lichtheimia corymbifera*	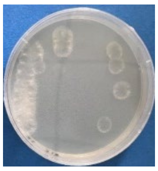	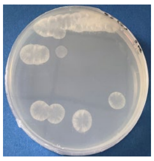
Aspergillus niger	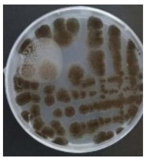	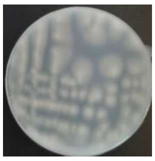	*Trichosporon asahii*	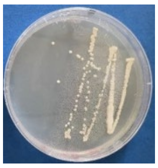	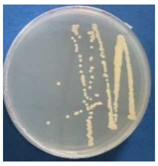
Trichosporon asahii	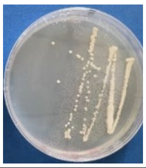	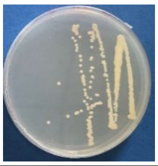			

Supplementary Figure 4: Validation of macromolecule degradation ability of representative microbial strains on solid media.

Strain	（D）	（d）	d/D	（D）	（d）	d/D
	Cellulose				Protein	
Puer_*Bh*	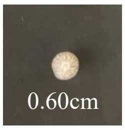	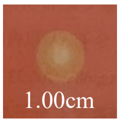	1.67	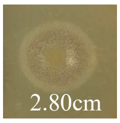	0	0
Puer_*Bl*	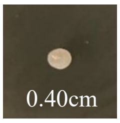	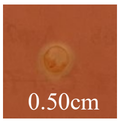	1.25	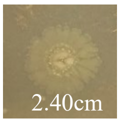	0	0
Puer_*Bs*	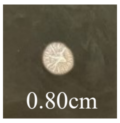	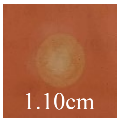	1.34	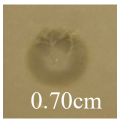	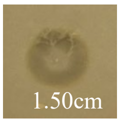	2.14
Puer_*Ba*	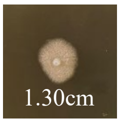	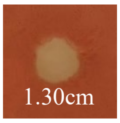	1.00	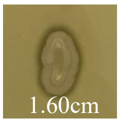	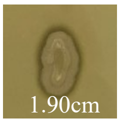	1.19
	Starch				Pectin	
Puer_*Bh*	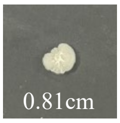	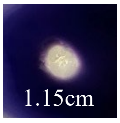	1.42	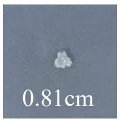	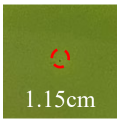	1.42
Puer_*Bl*	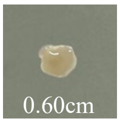	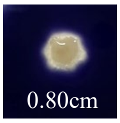	1.33	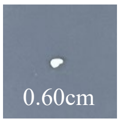	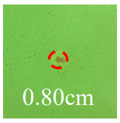	1.33
Puer_*Bs*	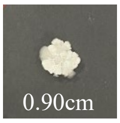	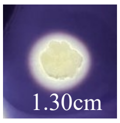	1.44	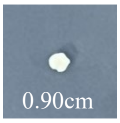	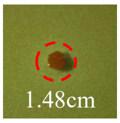	1.64
Puer_*Ba*	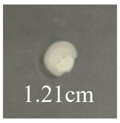	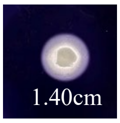	1.15	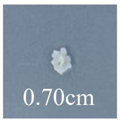	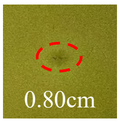	1.14

Supplementary Figure 4 (Continued)

Strain	（D）	（d）	d/D	（D）	（d）	d/D
	Pectin			Protein		
Puer_*At*	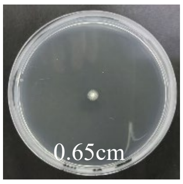	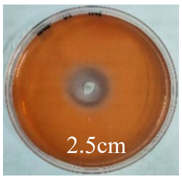	3.85	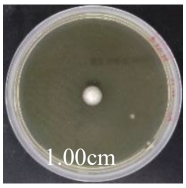	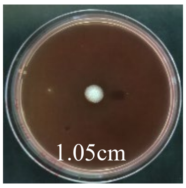	1.05
Puer_*An*	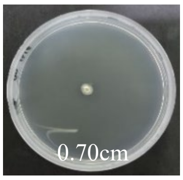	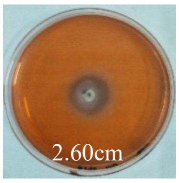	3.71	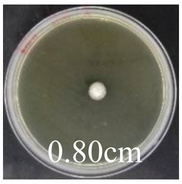	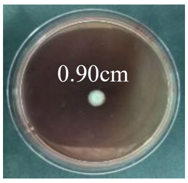	1.13
	Starch			Protein		
Puer_*At*	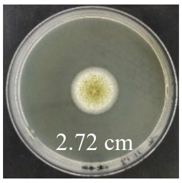	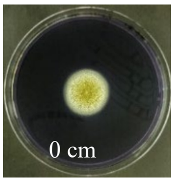	0	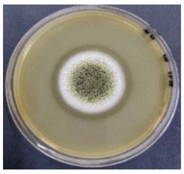	d=3.6 cm<br>D=4.15 cm	1.15
Puer_An	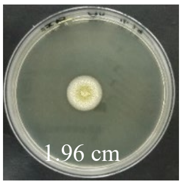	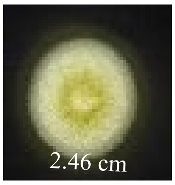	1.26	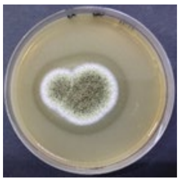	d=2.90 cm<br>D=4.00 cm	1.38

Notes: Agar plate hydrolysis assays were conducted using carboxyl methyl cellulose, skim milk powder, starch, and pectin as substrates. D: hydrolysis ring diameter; d: colony diameter; D/d ratio > 1 indicates enzymatic activity.
